# Chemically Synthesized Glycosides of Hydroxylated Flavylium Ions as Suitable Models of Anthocyanins: Binding to Iron Ions and Human Serum Albumin, Antioxidant Activity in Model Gastric Conditions

**DOI:** 10.3390/molecules191220709

**Published:** 2014-12-11

**Authors:** Sheiraz Al Bittar, Nathalie Mora, Michèle Loonis, Olivier Dangles

**Affiliations:** 1 University of Avignon, INRA, UMR408, Avignon 84000, France; E-Mails: sheiraz.al-bittar@alumni.univ-avignon.fr (S.A.B.); Nathalie.Mora@univ-avignon.fr (N.M.); 2 INRA, University of Avignon, UMR408, Avignon 84000, France; E-Mail: michele.loonis@avignon.inra.fr

**Keywords:** anthocyanin, 3-deoxyanthocyanidin, flavylium, chalcone, iron, lipid peroxidation, serum albumin

## Abstract

Polyhydroxylated flavylium ions, such as 3',4',7-trihydroxyflavylium chloride (P1) and its more water-soluble 7-*O*-β-d-glucopyranoside (P2), are readily accessible by chemical synthesis and suitable models of natural anthocyanins in terms of color and species distribution in aqueous solution. Owing to their catechol B-ring, they rapidly bind Fe^III^, weakly interact with Fe^II^ and promote its autoxidation to Fe^III^. Both pigments inhibit heme-induced lipid peroxidation in mildly acidic conditions (a model of postprandial oxidative stress in the stomach), the colorless (chalcone) forms being more potent than the colored forms. Finally, P1 and P2 are moderate ligands of human serum albumin (HSA), their likely carrier in the blood circulation, with chalcones having a higher affinity for HSA than the corresponding colored forms.

## 1. Introduction

Anthocyanins are responsible for the colors of numerous flowers, fruits, vegetables and even cereals. Colors expressed by anthocyanins vary from red to blue depending on pH, self-association (especially, in the case of acylated anthocyanins) and interactions with metal ions (Al^3+^, Fe^3+^, Mg^2+^) and phenolic copigments, such as flavones, flavonols and hydroxycinnamic acids [1,2,3,4,5]. Through their coloring properties, anthocyanins strongly contribute to food quality and appeal to consumers. They may also contribute to the health benefits of diets rich in plant products [6]. For instance, anthocyanins with an electron-rich B-ring, in particular an *o*-dihydroxylated B-ring (catechol), are intrinsically good antioxidants, either by acting as electron donors to reactive oxygen species or by chelating transition metal ions (potential inducers of oxidative stress) as inert complexes [7,8].

Dietary anthocyanins can be partly absorbed along the gastrointestinal (GI) tract (from stomach to colon) [9] but have an overall poor bioavailability in humans, at least based on the very low circulating concentrations of the native forms and their conjugates [10]. In fact, anthocyanins may be relatively unstable in the intestine [11,12,13,14] and, as polyphenols in general [15], undergo an extensive catabolism by intestinal glucosidases and by the enzymes of the colonic microbiota. In particular, hydrolysis of the anthocyanins’ glycosidic bond at C3-OH, which releases highly unstable anthocyanidins, must be a critical step toward cleavage of the C-ring. Consequently, a large part of the health benefits of anthocyanins is expected to be mediated by their degradation products and their conjugates [16].

On the other hand, anthocyanins, as ubiquitous dietary polyphenols, can accumulate under their native form in the GI tract and possibly protect dietary lipids and proteins against oxidation. Indeed, in gastric conditions (high O_2_ content, acidic pH), lipid peroxidation induced by dietary heme iron could be very significant but efficiently inhibited by polyphenols [17,18,19,20,21]. Through reduction of high-valence heme iron, polyphenols could preserve the nutritional value of the dietary bolus and prevent the formation of toxic lipid peroxidation products. This hypothesis of an early antioxidant protection by dietary polyphenols, including anthocyanins, is gaining evidence from *in vivo* studies [22].

Once in the general blood circulation, polyphenols and their metabolites, typically bound to human serum albumin (HSA) [23,24], are delivered to tissues for specific biological effects [15].

3-Deoxyanthocyanidins and their glucosides have been identified in cereals such as red sorghum [25]. Lacking the C3-OH group of anthocyanidins, which is critically involved in their degradation, 3-deoxyanthocyanidins express more stable colors [26]. They are also promising pigments in terms of potential health benefits, expressed by antioxidant and cell-specific effects [27,28,29]. So far, little is known about their bioavailability but it may be speculated that it is higher than that of anthocyanins, as 3-deoxyanthocyanidins are probably less prone to catabolism in the GI tract. For future development as food ingredients, it is also noteworthy that mutagenesis-assisted breeding can dramatically increase 3-deoxyanthocyanidin accumulation in sorghum leaves [30].

**Figure 1:**
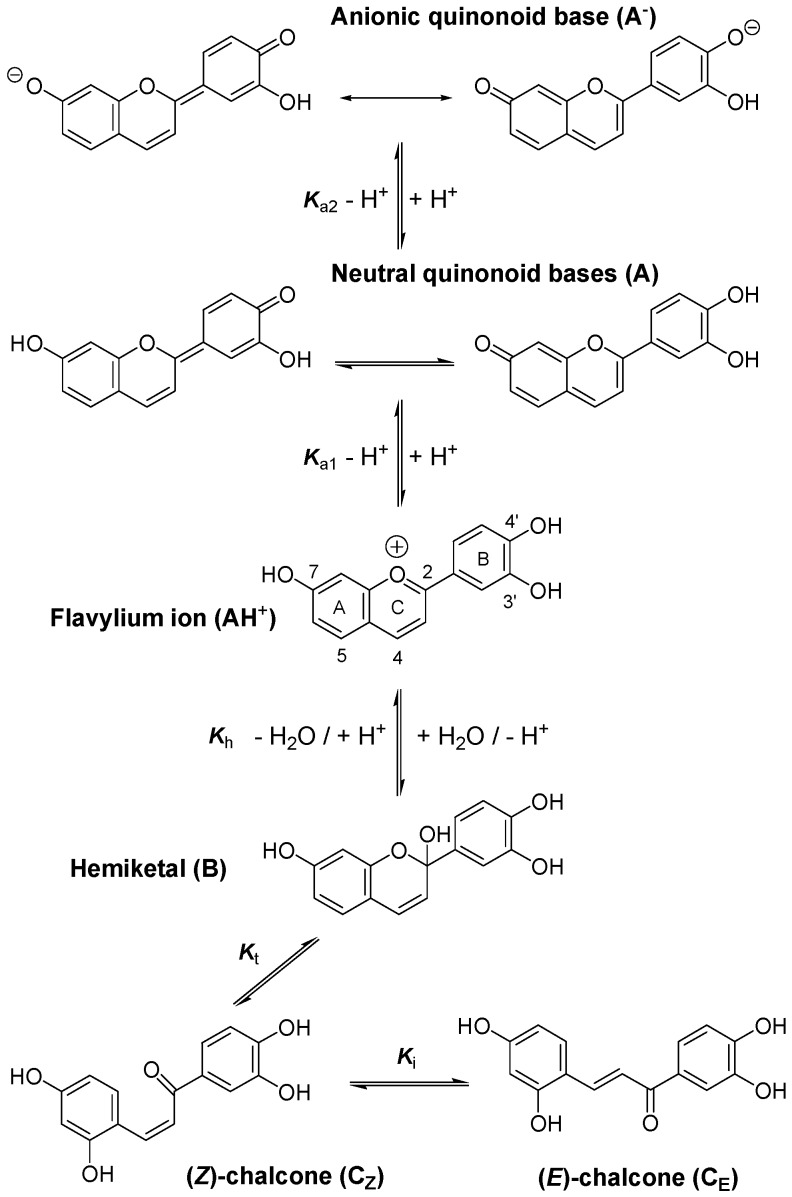
Structural transformations of the 3',4',7-trihydroxyflavylium ion (P1).

Interestingly, 3-deoxyanthocyanidins and their glucosides, in particular simplified analogs lacking the C5-OH group, are far more accessible by chemical synthesis than even the simplest anthocyanins. In a previous paper [31], we reported the chemical synthesis, structural transformations, aluminium binding and radical-scavenging (DPPH test) of 3',4',7-trihydroxyflavylium chloride (P1) and its 7-*O*-β-d-glucoside (P2) (Figure 1). In this work, their capacity to bind iron ions and inhibit heme-induced lipid peroxidation in mildly acidic conditions (a model of postprandial oxidative stress in the stomach) will be quantitatively studied as well as their affinity for HSA, their likely carrier in the blood circulation. In each model, the activity of the colored and colorless forms will be discriminated.

The aim of this work is to emphasize, through detailed quantitative physico-chemical analyses, that readily available 3-deoxyanthocyanidins—a relatively overlooked class of natural pigments—are interesting colorants and antioxidants deserving further examination for future applications.

## 2. Results and Discussion

As a general comment, interpretation of our data rests on the well-established scheme of structural transformation for the flavylium ion of anthocyanins (Figure 1) [32,33]. However, flavylium ions lacking the glycosyloxy substituent of natural anthocyanins at C3 display some peculiarities: dehydration of hemiketal B into the highly planar flavylium ion is faster as well as its sequential conversion into C_Z_ and C_E_. C_E_ is also much more stable than C_Z_ (*K*_i_ ≈ 75 for P2 [32]) whereas the two isomers display close stability with natural anthocyanins. Consequently, B and C_Z_ can be regarded as transient (non-accumulating) intermediates in the overall conversion of the flavylium ion into the corresponding (*E*)-chalcone.

### 2.1. Iron-Pigment Binding

Together with copigmentation and self-association, metal-anthocyanin binding is one of the most important mechanisms for varying and stabilizing natural colors [1]. In our previous work [31], both P1 and P2 were shown to bind Al^III^ in mildly acidic solutions, thereby forming chelates having a quinonoid chromophore as the result of the simultaneous loss of the two protons at C3'-OH and C4'-OH. Interestingly, the Al^III^-P2 complex is more resistant than the Al^III^-P1 complex toward water addition leading to the free (unbound) (*E*)-chalcone.

In this work, P1 and P2 are compared for their ability to bind Fe^III^ and Fe^II^. As iron ions take part in the production of reactive oxygen species (e.g., via the Fenton reaction [34]), their binding as redox-inert chelates can be considered a potential antioxidant mechanism. Moreover, transition metal ions such as iron and copper ions being present in our diet [35], metal-anthocyanin binding could also take place in the upper GI tract (in mildly acidic conditions) and modulate the properties and stability of anthocyanins in this biological site.

#### 2.1.1. Pigment P1

**Figure 2:**
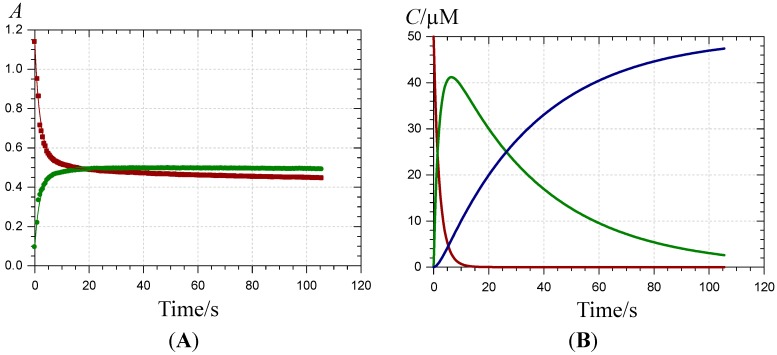
Kinetics of Fe^III^-P1 binding (pH 4 acetate buffer, 25 °C, 4 equiv. Fe^III^). (**A)** Time-dependence of the visible absorbance at 470 (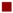) and 620 nm (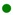); (**B**) time-dependence of the free pigment (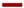) and the kinetic (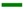) and thermodynamic (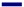) complexes.

**Figure 3:**
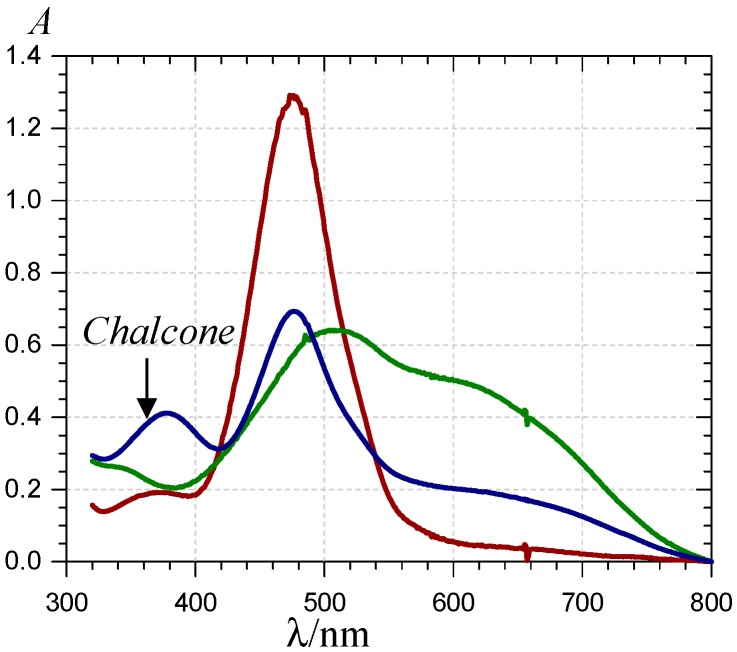
UV-visible spectra of P1 (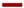), the P1-Fe^III^ complex (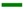, *ca*. 30 s after addition of 2 equiv. Fe^III^) and the complex formed *ca*. 400 s after addition of 5 equiv. Fe^II^ (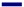) (pH 4 acetate buffer, 25 °C, pigment concentration = 50 µM).

The successive addition of P1 and Fe^III^ (0.5–5 equiv.) to a pH 4 acetate buffer results in the fast decay of A(470 nm) and the development of a broad visible band in the range 450–750 nm with an absorption maximum at *ca*. 510 nm (Figure 2 and Figure 3). Those spectral changes can be interpreted by the formation of a P1-Fe^III^ complex having a quinonoid chromophore that acts as a donor in a charge transfer interaction with the Fe^III^ empty orbitals.

Over 2 min, free chalcone formation (typical absorption at λ_max_ = 375 nm) is negligible (confirmed by HPLC-MS analysis), even when P1 is in excess (0.5 equiv. Fe^III^). When compared with Al^III^-P1 binding [31], Fe^III^-P1 binding is much faster and quasi-irreversible as the final maximal absorbance at 620 nm is reached with 1 equiv. Fe^III^. The time dependence of A(470 nm) and A(620 nm) can be interpreted by the fast formation of a first complex (rate constant of binding *k*_b_) followed by its slower first-order conversion into a second complex (rate constant of rearrangement *k*_r_) (Figure 2). A simultaneous curve-fitting of both curves (Equations (1)–(3)) gives access to the corresponding rate constants and molar absorption coefficients (Table 1).

(1) $$-\frac{\text{d}}{\text{dt}}{\text{[Fe}}^{\text{III}}\text{]}=-\frac{\text{d}}{\text{dt}}\text{[L]}=k_{\text{b}}{\text{[Fe}}^{\text{III}}\text{][L]}$$

(2) $$\frac{\text{d}}{\text{dt}}{\text{[Fe}}^{\text{III}}L_{1}]=k_{\text{b}}{\text{[Fe}}^{\text{III}}\text{][L]}-k_{\text{r}}{\text{[Fe}}^{\text{III}}L_{1}]$$

(3) $$\frac{\text{d}}{\text{dt}}{\text{[Fe}}^{\text{III}}L_{2}]=k_{\text{r}}{\text{[Fe}}^{\text{III}}L_{1}]$$

The *k*_r_ values, which suggest a quasi-total consumption of the first complex over 2 min, are much higher than those obtained for water addition to free P1 (chalcone formation) and its Al^III^ complex [31]. Moreover, at the end of the kinetics, the broad absorption band, almost covering the visible spectrum and still well visible after several hours, is not compatible with a Fe^III^-chalcone complex. Addition of Fe^III^ (5 equiv.) to an equilibrated solution of P1 in which C_E_ is the dominant species shows the binding of the minor colored forms with little impact on the chalcone band over 2 min (data not shown), thus indicating that C_E_ does not bind Fe^III^ in mildly acidic solution. The hypothesis of Fe^III^ reduction and concomitant oxidation of P1 is also not consistent with the spectrum obtained after acidification to pH < 2 (total recovery of free P1) and the HPLC-MS analysis (no oxidation product detected). Finally, one can propose the formation of a kinetic product (complex 1) evolving into a thermodynamic product (complex 2), possibly by additional coordination of acetate ions. Similar kinetic patterns were previously observed with other phenols in their binding to Fe^III^ [36]. Thus, starting from the flavylium ion, addition of Fe^III^ results in the fast binding of the colored forms (in fast acid-base equilibrium, collectively noted L in Equations (1)–(3)). Concomitantly, the fraction of free flavylium in solution is greatly lowered so that water addition (and subsequent chalcone formation) is quenched.

**Table 1:** Kinetic analysis of P1-Fe^III^ binding. Simultaneous curve-fitting of the A(470 nm) and A(620 nm) *vs.* time curves according to a simple model assuming irreversible 1:1 binding (rate constant *k*_b_) followed by first-order rearrangement of complex 1 into complex 2 (rate constant *k*_r_) (pH 4 acetate buffer, 25 °C, pigment concentration = 50 µM).

M_t_/L_t_, λ/nm	*k*_b_/M^−1^·s^−1^	10^3^ *k*_r_/s^−1^	ɛ_1_/M^−1^·cm^−1^	ɛ_2_/M^−1^·cm^−1^
1, 470	17,890 (±180)	4 (±2)	10,340	7910
620			12,270	10,810
2, 470	7190 (±250)	44 (±4)	13,190	10,320
620			12,180	12,780
3, 470	4450 (±60)	16 (±3)	12,010	10,300
620			13,160	13,060
4, 470	2670 (±30)	29 (±2)	10,360	8900
620			9710	9930
5, 470	3370 (±50)	59 (±2)	12,830	10,300
620			10,680	11,580
5, 470 ^a^	250 (±30)	-	10,700	-
630			11,800	
375			4200	

Notes: ^a^ Fe^II^, apparent first-order autoxidation of Fe^II^: *k*_autox_ = 58 (±6) × 10^−5^ s^−1^; chalcone formation: *k*_h_^obs^ = 95 (±4) × 10^−5^ s^−1^, ɛ_CE_ = 33,800 M^−1^·cm^−1^ at 375 nm.

**Figure 4:**
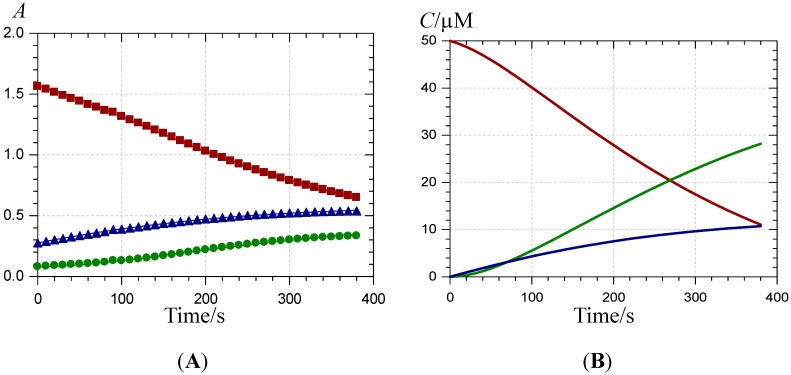
Kinetics of Fe^II^–P1 binding (pH 4 acetate buffer, 25 °C, 5 equiv. Fe^II^). (**A**) time-dependence of the visible absorbance at 470 (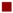), 620 (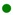) and 375 nm (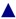); (**B**) time-dependence of the free pigment (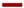), the metal complex (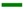) and the free chalcone (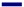).

When Fe^II^ is added in an equimolar concentration, a slow decay of A(470 nm) paralleled by a slow increase of A(375 nm) is observed. As the corresponding absorption bands are not shifted in comparison to free P1, it can be concluded that Fe^II^-P1 binding is negligible and the spectral changes are fully ascribed to water addition to free P1 with concomitant chalcone formation. A double first-order curve-fitting at 470 and 375 nm yields: *k*_h_^obs^ = 140 (±1) × 10^−5^ s^−1^, in reasonable agreement with the value in the absence of Fe^II^ (*k*_h_^obs^ ≈ 120 × 10^−5^ s^−1^, half-life of free P1 at pH 4 ≈ 10 min). However, addition of an excess Fe^II^ (5 equiv.) causes the slow development of a broad visible band in the range 500–750 nm, again with no shift in the band at 470 nm (in contrast to Fe^III^, see Figure 3 and Figure 4). Moreover, a relatively fast accumulation of free chalcone reaching saturation after 300–400 s is also observed. In a pH 4 acetate buffer, Fe^II^ titration (ferrozine test, data not shown) shows that Fe^II^ autoxidation is negligible. However, the broad visible band appearing in the range 500–750 nm is evidence for the formation of a Fe^III^-P1 complex [36,37]. Thus, it is proposed that a weak Fe^II^–P1 binding occurs that promotes a slow Fe^II^ autoxidation (apparent first-order rate constant *k*_autox_) without totally quenching water addition to P1. Then, the Fe^III^–P1 slowly accumulates. Using this kinetic model (detailed below with P2), the corresponding rate constants can be estimated (Table 1) and the different concentrations plotted as a function of time (Figure 4).

#### 2.1.2. Pigment P2

**Figure 5:**
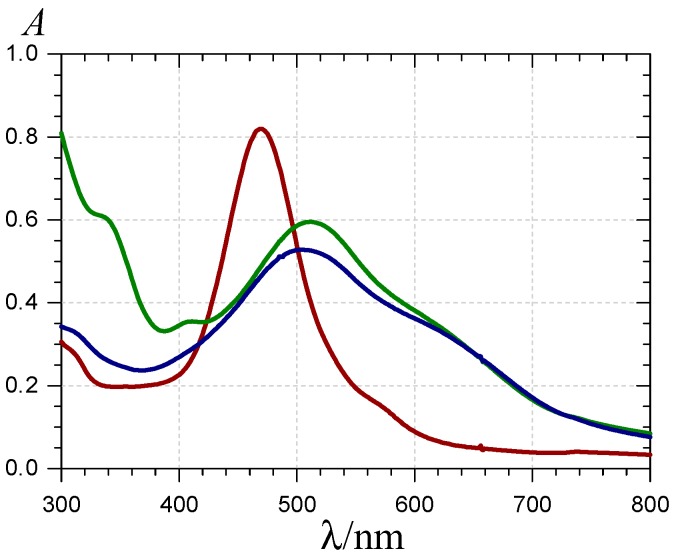
UV-visible spectra of P2 (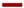), the P2-Fe^III^ complex (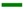, *ca*. 10 s after Fe^III^ addition) and the complex formed *ca*. 10 min after addition of Fe^II^ (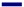) (pH 4 acetate buffer, 25 °C, pigment concentration = 50 µM, iron-P2 molar ratio = 5).

The successive addition of P2 and Fe^III^ (0.5–5 equiv.) to a pH 4 acetate buffer results in spectral changes (Figure 5) that are close to the ones observed with P1. They are consistent with the formation of a P2-Fe^III^ complex having a quinonoid chromophore that acts as a donor in a charge transfer interaction with Fe^III^.

Unlike Al^III^ [31], Fe^III^ binds P2 even more rapidly than P1, so that an accurate kinetic analysis is not possible by conventional UV-visible spectroscopy. However, assuming irreversible 1:1 binding, a lower limit can be proposed for the second-order rate constant of P2-Fe^III^ binding: *k*_b_ > 5 × 10^3^ M^−1^·s^−1^. Free chalcone formation is negligible (confirmed by HPLC-MS analysis), even when P2 is in excess (0.5 equiv. Fe^III^). However, a slight decay of A(650 nm) is observed with 0.5–1 equiv. Fe^III^. Although fast, Fe^III^-P2 binding is reversible and the final maximal absorbance at 650 nm is only reached with an excess Fe^III^ (*ca*. 5 equiv.).

The plot of Δ*A* = *A*_max_ − *A*_0_ (at 650 nm) as a function of the total metal concentration M_t_ can be successfully analyzed according to a 1:1 reversible binding model (Equations (4) and (5)), thereby allowing the determination of the Fe^III^-P2 binding constant: *K*_b_ = 21 (±6) ×10^3^ M^−1^, Δɛ = ɛ_complex_ − ɛ_P2_ = 6500 (±500) M^−1^·cm^−1^ (*r* = 0.995). This *K*_b_ value is identical to the one estimated for the Al^III^-P2 complex [31]. Thus, the two trivalent hard metal cations Fe^III^ and Al^III^ have the same affinity for the P2 catechol nucleus. However, the Fe^III^-P2 binding is much faster, the equilibrium being reached in a few seconds *vs.* several minutes with Al^III^.

(4) $$\Delta A=\Delta \varepsilon {\text{(Fe}}_{\text{total}}^{\text{III}}-[{\text{Fe}}^{\text{III}}])$$

(5) $${\text{Fe}}_{\text{total}}^{\text{III}}=[{\text{Fe}}^{\text{III}}](1+\frac{K_{\text{b}}L_{\text{total}}}{1+K_{\text{b}}[{\text{Fe}}^{\text{III}}]})$$

*L*_total_: total ligand concentration, ${\text{Fe}}_{\text{total}}^{\text{III}}$ : total metal concentration, *K*_b_: metal-pigment binding constant, ∆ε = ε_FeL_^650^ − ɛ_L_^650^.

**Figure 6:**
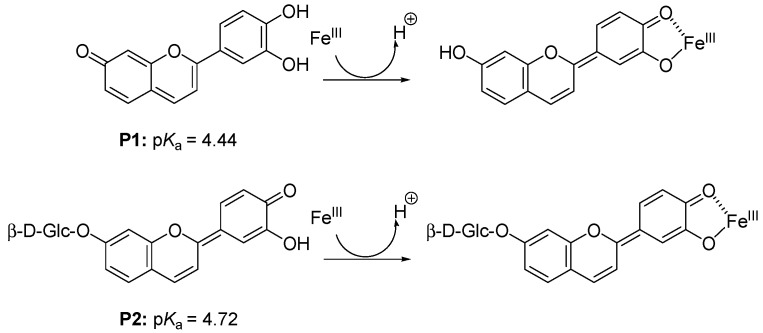
Iron-pigment binding.

The observation that Fe^III^-P2 binding is faster than Fe^III^-P1 binding may be ascribed to different binding species in solution at pH 4. Indeed, the higher acidity of the P1 flavylium ion [31] probably indicates that P1 deprotonation at C7-OH is more favorable than at C4'-OH while P2 deprotonation can only occur at C4'-OH (Figure 6). Thus, Fe^III^-P1 binding probably requires a thermodynamically unfavorable change in quinonoid tautomer that is not needed with P2.

Like P1, P2 apparently binds Fe^II^ much more slowly than Fe^III^. For instance, while Fe^III^-P2 binding reaches equilibrium in a few seconds, Fe^II^-P2 binding requires *ca*. 4 min with 5 equiv. Fe^II^ (Figure S1). With 1 equiv. Fe^II^, the equilibrium is not even achieved after 10 min. Interestingly, with 5 equiv. iron, the final spectra characteristic of the complexes are very close, except for a strong absorption band developing below 360 nm for the Fe^III^-P2 complex (shoulder at 340 nm) that is characteristic of free Fe^III^ (Figure 4). It can thus be proposed that the same Fe^III^-P2 complex is ultimately formed after addition of Fe^III^ or Fe^II^. In other words, Fe^II^ slowly binds P2 with simultaneous conversion into Fe^III^, while free Fe^II^ in excess remains stable in solution. In particular, the broad absorption band beyond 600 nm (not observed with the Al^III^-P2 complex) is characteristic of a catechol-to-Fe^III^ charge transfer interaction.

The curves showing the time dependence of A(470 nm) and A(650 nm) display short lag phases (Figure S1) suggesting that a preliminary slow autoxidation of Fe^II^ (apparent first-order rate constant *k*_autox_) must take place to trigger the binding (second-order rate constant *k*_b_). Hence, both curves could be fitted against the following model (Equations (6)–(10), Table 2).

(6) $$-\frac{\text{d}}{\text{dt}}{\text{[Fe}}^{\text{II}}\text{]}=k_{\text{autox}}{\text{[Fe}}^{\text{II}}]$$

(7) $$\frac{\text{d}}{\text{dt}}{\text{[Fe}}^{\text{III}}\text{]}=k_{\text{autox}}{\text{[Fe}}^{\text{II}}\text{]}-k_{\text{b}}{\text{[Fe}}^{\text{III}}\text{][L}]$$

(8) $$-\frac{\text{d}}{\text{dt}}\text{[L}]=k_{\text{b}}{\text{[Fe}}^{\text{III}}\text{][L}]+k_{\text{h}}^{\text{obs}}\text{[L]}$$

(9) $$\frac{\text{d}}{\text{dt}}{\text{[LFe}}^{\text{III}}]=k_{\text{b}}{\text{[Fe}}^{\text{III}}\text{][L}]$$

(10) $$\frac{\text{d}}{\text{dt}}{\text{[C}}_{\text{E}}\text{]}=k_{\text{h}}^{\text{obs}}\text{[L]}$$

**Table 2:** Kinetic analysis of the spectral changes following addition of Fe^II^ to a P2 solution (pH 4 acetate buffer, 25 °C, pigment concentration = 50 µM).

M_t_/L_t_, λ/nm ^a^	10^5^*k*_autox_/s^−1^	*k*_b_/M^−1^·s^−1^	ɛ_ML_/M^−1^·cm^−1^
0.5, 470 (*r* = 0.9978)	215 (±2)	n.d. ^b^	8800 ^c^
650 (*r* = 0.9975)	13.7 (±0.2) ^d^		7200 ^c^
1, 470 (*r* = 0.9985)	169 (±1)	n.d. ^b^	8800 ^c^
650 (*r* = 0.9985)	13.3 (±0.4) ^d^		7200 ^c^
2, 470 (*r* = 0.9992)	181 (±5)	473 (±34)	8870
650 (*r* = 0.9993)			7320
3, 470 (*r* = 0.9998)	154 (±2)	663 (±29)	8850
650 (*r* = 0.9996)			7110
4, 470 (*r* = 0.9998)	141 (±2)	593 (±23)	8670
650 (*r* = 0.9999)			7070
5, 470 (*r* = 0.9988)	163 (±7)	785 (±75)	8810
650 (*r* = 0.9994)			7340

Notes: ^a^ Each *A vs.* time curve is a mean of 2 experimental curves; ^b^ Steady-state assumed for Fe^III^; ^c^ Set constant; ^d^ Apparent rate constant of water addition (*k*_h_^obs^).

With an excess Fe^II^, chalcone formation can be neglected with P2 (*k*_h_^obs^ = 0), while it is detectable with P1 (Figure 3 and Figure 4).

In summary, Fe^III^ rapidly binds both P1 and P2 in mildly acidic solutions, thereby quenching their conversion into the corresponding chalcones. With P2, the binding is faster but reversible. By contrast, P1 and P2 only weakly interact with Fe^II^, thereby promoting its autoxidation with subsequent fast binding of Fe^III^.

### 2.2. Pigment-Serum Albumin Binding

HSA, the major plasma protein (*ca*. 0.6 mM), is responsible for the transport of a large variety of ligands [38], including drugs and dietary components such as fatty acids and polyphenols [23,24,39]. The heart-shaped structure of HSA consists of three helical domains I (1–195), II (196–383) and III (384–585), each being divided into sub-domains A and B [38]. The main binding sites of drugs and polyphenols are site 1 and site 2 (respectively located in sub-domains IIA and IIIA), which consist in hydrophobic pockets lined by positively charged aminoacid residues (Arg, Lys).

Whereas glycoside hydrolysis prior to intestinal absorption seems the rule with polyphenols in general, native anthocyanins (glycosides) have been detected in the blood circulation, although in very low (sub-micromolar) concentration [10]. Moreover, under physiological conditions, delphinidin, cyanidin and pelargonidin 3-*O*-β-d-glucosides have been reported to bind to HSA site 1 with thermodynamic binding constants in the range 69–144 × 10^3^ M^−1^ [40]. So far, no work has discriminated the colored and colorless forms by their affinity for HSA, despite the fact that the colorless forms are expected to largely prevail at equilibrium in neutral conditions.

**Figure 7:**
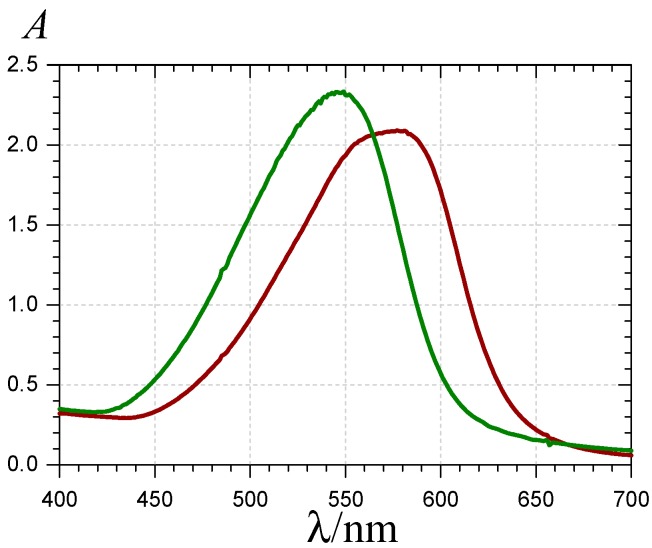
UV-visible spectra of P1 (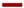) and the P1-HSA complex (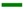) (pH 7.4 phosphate buffer, 25 °C, pigment concentration = 50 µM, HSA-P1 molar ratio = 2).

In this study, pigment–HSA binding was first evidenced by UV-visible spectroscopy. For instance, the visible band of P1 at pH 7.4 shifts from 540–570 nm when an excess HSA (2 equiv.) is added (Figure 7). However, this is not so with P2 (unchanged λ_max_ = 530 nm). The bathochromic shift specifically observed for P1 suggests a role for the free C7-OH group. At pH 7.4, the anionic quinonoid form makes a substantial contribution. In the case of P1, the binding to HSA could even favor the formation of the anionic quinonoid base, in agreement with the high density of positive charges (protonated Lys and Arg residues) present in sub-domain IIA, the typical binding site of flavonoids [39]. To check this hypothesis, the pH dependence of the visible spectrum of P1 around neutrality was evaluated in the presence and absence of HSA. Very similar titration curves were obtained in agreement with a p*K*_a2_ value of *ca*. 7.1 (Table 3, Figure S2). Thus, binding to HSA does not significantly shift the equilibrium between the neutral and anionic quinonoid bases. Hence, the HSA-induced bathochromic shift may be rather ascribed to perturbation in the molecular orbitals specifically involved in the visible band, e.g., the HOMO of the anionic quinonoid base (due to possible charge transfer interactions) with no impact on the global stability.

**Table 3:** Kinetic and thermodynamic parameters for the structural transformations of P1 and P2 in neutral conditions with and without HSA.

	P1	P2
p*K*_a2_, *r*_a_ (550 nm), no HSA	7.12 (±0.05), 6.3 (±0.6) ^a^	n.a. ^b^
p*K*_a2_, *r*_A_ (580 nm), 5 equiv. HSA	7.11 (±0.04), 3.1 (±0.1) ^a^	n.a. ^b^
*k*_h_^obs^ (s^−1^), 530 nm, no HSA	n.a., too slow *ca*. −10% color loss after 45 min	88 (±1) × 10^−5^ ^c^
*k*_h_^obs^ (s^−1^), 530 nm, 2 equiv. HSA	n.a., too slow *ca*. −10% color loss after 45 min	81 (±1) × 10^−5^ ^c^

Notes: ^a^ From the curve-fitting of the *A vs.* pH curves at equilibrium (*r*_A_ = ratio of the molar absorption coefficients of the anionic to neutral quinonoid bases); ^b^ No proton loss in the pH range 6–8, total conversion of colored forms into chalcone; ^c^ From a first-order curve-fitting of the color loss at pH 7.4.

After equilibration for *ca*. 24 h, the titration curves were modified by the gradual appearance of the chalcone (Figure S2). The residual color is approximately the same in the absence or presence of HSA. Thus, HSA has a minor impact on the quinonoid bases-chalcone equilibrium, which is indicative that the different forms have close affinities for the protein. The residual color at pH 7.4 is consistent with a *K*_i_ = (C_E_)/(A) value of *ca*. 10, in agreement with the p*K*_a1_ and p*K*’_h_ values previously determined for P1 ([31], 4.44 and 3.45, respectively). From the *K*_i_ and *K*_a2_ values, a distribution diagram of the different P1 species can be plotted around neutrality in the presence or absence of HSA (Figure S3).

In the case of P2, the situation is simpler as no anionic quinonoid base can form. Monitoring the decay of the color over time shows that the apparent first-order rate constant of water addition (*k*_h_^obs^) is only weakly affected by HSA (Table 3). Moreover, whether HSA is present or not, the color loss can be considered complete. Thus, the quinonoid base concentration at equilibrium is negligible (*K*_i_ > 10).

**Figure 8:**
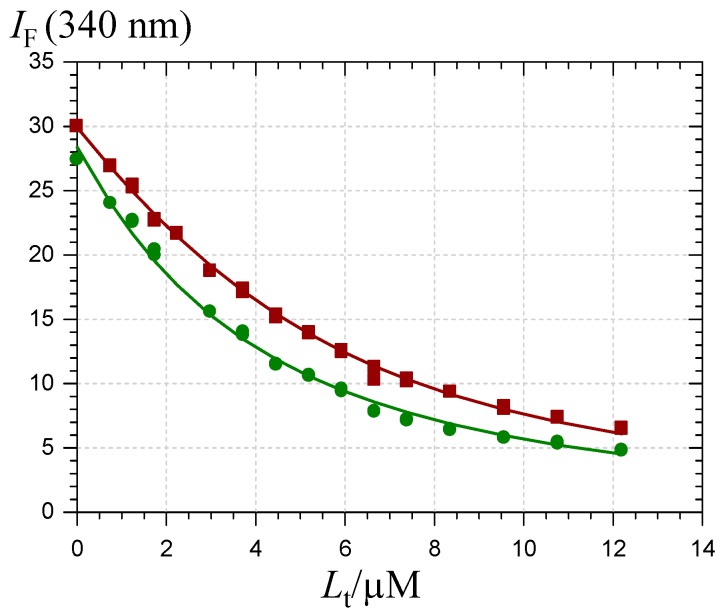
Quenching of the HSA fluorescence by the P1 quinonoid bases (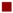) and chalcone (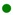). HSA concentration = 2 µM, pH 7.4 phosphate buffer, 37 °C, excitation at 295 nm.

For an accurate estimation of the corresponding binding constants, the pigment-HSA binding was investigated by fluorescence spectroscopy. The intrinsic HSA fluorescence at 340 nm (excitation at 295 nm) is due to its single Trp residue (Trp-214) located in sub-domain IIA. Its strong quenching by P1, P2 and their chalcones (Figure 8) is evidence that the binding actually takes place to or near this site.

The excitation wavelength was selected so as to maximize the fluorescence of the single Trp residue of HSA. However, the pigments, especially in their chalcone form, substantially absorb light at the excitation (295 nm) or/and emission (340 nm) wavelengths so that an inner filter correction is necessary. Hence, the protein fluorescence intensity is expressed in Equation (11).

(11) $$I_{\text{F}}=f_{\text{P}}\left[\text{P}\right]\text{ exp}\left(-\varepsilon_{\text{L}}l\;L_{\text{t}}\right)$$

(12) $$L_{\text{t}}=\;\left[\text{L}\right]\;\left(\text{1 }+K_{\text{b}}\left[\text{P}\right]\right)$$

(13) $$P_{\text{t}}=\;\left[\text{P}\right]\;\left(\text{1 }+K_{\text{b}}\left[\text{L}\right]\right)$$

In Equation (11), ƒ_P_ is the molar fluorescence intensity of HSA and ε_L_ stands for the sum of the molar absorption coefficients of the ligand at the excitation and emission wavelengths (Table 4). Its value is determined independently by UV-visible spectroscopy from a Beer’s plot. Finally, *l* is the mean distance travelled by the excitation light at the site of emission light detection. For the spectrometer used in this work, *l* is estimated to be 0.65 cm. Beside the expression of *I*_F_, the relationships used in the curve-fitting procedures were combinations of the mass law for the complex and mass conservation for the ligand L (pigments) and protein P (Equations (12) and (13), *L*_t_: total ligand concentration, *P*_t_: total protein concentration).

The *K*_b_ values (Table 4) illustrate two major points:

(1) The Glc moiety strongly destabilizes the complexes, especially for the colored forms (*K*_b_ value reduced by a factor 15–16).
(2) The chalcones, with their open more linear structure, display a higher affinity for HSA (*K*_b_ raised by a factor *ca*. 3 for P2) than the corresponding colored forms, although this increase is marginal with P1 in agreement with the investigation by UV-visible spectroscopy. This suggests that the very low circulating concentration of anthocyanins (in comparison to other flavonoids) [10,15] could be partly due to their conversion in colorless forms that may have escaped detection.

**Table 4:** Binding constant (*K*_b_) of pigments and their chalcones to HSA (2 µM) in a pH 7.4 phosphate buffer at 25 °C (*n* = 2).

	10^3^*K*_b_/M^−1^	10^6^*f*_P_/M^−1^	ε_L_/M^−1^ cm^−1^ ^a^	*r*
P1 colored forms	273 (±7)	15.5 (±0.1)	8900 + 5800	0.998
P1 chalcone	344 (±12) ^b^	14.2 (±0.1)	15,800 + 16,400 ^b^	0.997
P2 colored forms	17.5 (±0.5)	14.1 (±0.1)	3800 + 2800	0.999
P2 chalcone	58.4 (±1.9)	13.5 (±0.1)	7200 + 7000	0.998

Notes: ^a^ First value at 295 nm (excitation wavelength), second value at 340 nm (emission wavelength); ^b^ Apparent values including a minor contribution of the residual colored forms present at equilibrium. Assuming a 3:1 chalcone-to-colored forms molar ratio (see Figure S3), the true value for the sole chalcone can be estimated: *K*_b_ = 368 × 10^3^ M^−1^.

Interestingly, the *K*_b_ values for anthocyanidin 3-*O*-β-d-glucosides [40] are intermediates between the values for the P2 and P1 colored forms. Thus, P1 is a better HSA ligand than common anthocyanins, while the reverse is true for P2.

### 2.3. Inhibition of the Heme-Induced Peroxidation of Linoleic Acid

Given their poor bioavailability and extensive catabolism [10], anthocyanins are expected to exert their antioxidant activity in humans (in the restricted sense of electron donation to reactive oxygen species involved in oxidative stress) prior to intestinal absorption, *i.e*., in the gastro-intestinal tract, where they can accumulate in substantial concentrations and in their native forms following the consumption of plant products. On the other hand, in the gastric compartment, acidity, dioxygen and pro-oxidant species present in foods (iron, lipid hydroperoxides, H_2_O_2_) can provide suitable conditions for the oxidation of dietary polyunsaturated acids (PUFAs) in postprandial conditions [17,18,19,20,21,22]. This oxidation results in a loss of essential lipids and in the accumulation of potentially toxic lipid oxidation products. These lipid hydroperoxides and aldehydes can also alter dietary proteins and may even contribute to increasing the concentration of circulating minimally modified lipoproteins that are more prone to further oxidation and take part in atherogenesis. Based on simple *in vitro* models, our works suggest that heme-induced lipid oxidation is particularly fast in the first period of gastric digestion (pH 4–6) but efficiently inhibited by plant antioxidants (polyphenols, α-tocopherol, carotenoids) [21,41,42]. Recently, the pertinence of our model was confirmed by gastric fluid analysis in minipigs [22].

With linoleic acid (LH) as a model of dietary PUFA, conjugated dienes (CDs) are acceptable markers of the early phase of lipid oxidation and can be approximately identified with lipid hydroperoxides (LOOH), the corresponding alcohols (LOH) making only a minor contribution. CD accumulation is easily followed by UV-visible spectroscopy in the presence or absence of antioxidant.

**Figure 9:**
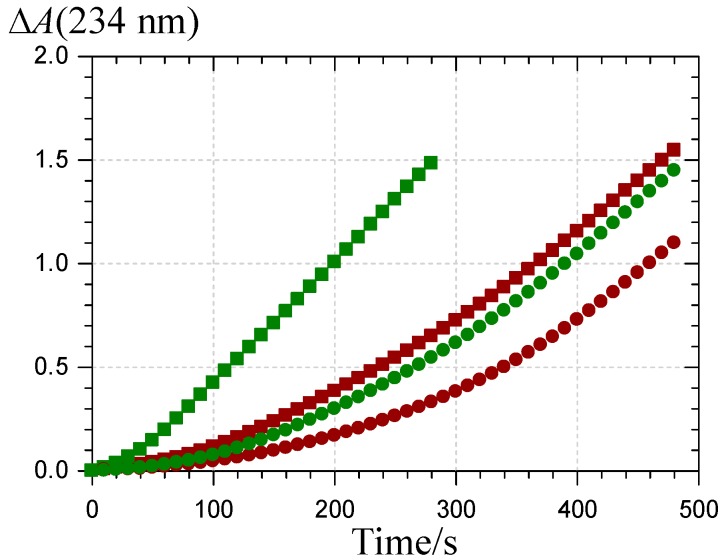
Inhibition of the metmyoglobin-induced peroxidation of linoleic acid. Pigment concentration = 2.5 µM, 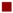: P1 colored forms, 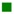: P2 colored forms, 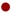: P1 chalcone, 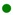: P2 chalcone (pH 5.8 phosphate buffer + Brij^®^35, 37 °C).

A simple visual comparison of the curves featuring CD accumulation in the presence of a fixed pigment concentration (Figure 9) shows that P2, whether in its colored or chalcone form, is a poorer antioxidant than P1, in agreement with our preliminary investigation of the DPPH radical-scavenging activity [31]. Interestingly, the chalcone forms come up as more potent inhibitors than the corresponding colored forms.

The metmyoglobin-induced peroxidation of linoleic acid is initiated via a Fe^III^-Fe^IV^ redox cycle involving small concentrations of PUFA hydroperoxides inevitably contaminating any PUFA sample [43,44,45]. As hydrophilic antioxidants, polyphenols typically inhibit lipid peroxidation at the initiation stage by reducing hypervalent heme iron (Fe^IV^), instead of significantly scavenging lipid peroxyl radicals, as lipophilic antioxidants (α-tocopherol, carotenoids) do [21,41,42,46].

**Figure 10:**
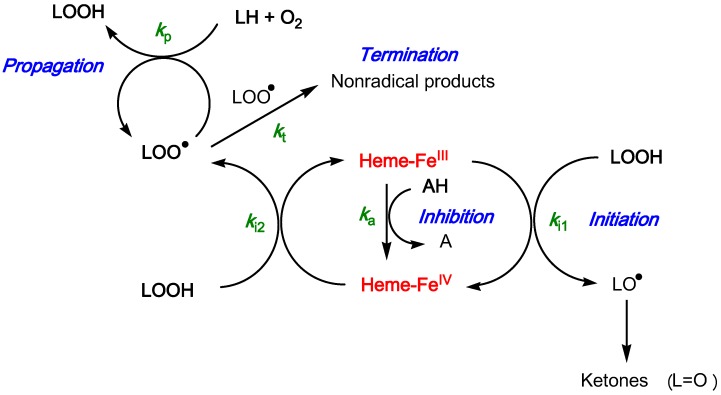
Metmyoglobin-induced peroxidation of linoleic acid and its inhibition by polyphenols (LH: PUFA, LOOH: PUFA hydroperoxide, AH: antioxidant, H^+^ and HO^−^ ions omitted).

The reactions involved in the heme-induced peroxidation of linoleic acid in the presence of an antioxidant are summed up in Figure 10 with the corresponding rate constants.

In the absence of antioxidant, the short lag phase is better reproduced without assuming a steady-state for Fe^IV^. On the other hand, the two initiation rate constants can be taken equal (*k_i1_* = *k_i2_*) so as to restrict the total number of adjustable parameters. Thus, in a first step, the curves of uninhibited lipid peroxidation are analyzed so as to estimate a value for *k*_i1_ (rate constant of LOOH cleavage by low-valence heme) that will be used in all curve-fitting experiments related to inhibited peroxidation with the following adjustable parameters (see Appendix for details): $r_{2}=\frac{k_{p}}{\sqrt{2k_{\text{t}}}}$, a measure of PUFA oxidizability, $\text{AE}=\frac{k_{\text{a}}}{k_{\text{i}2}}$, the antioxidant efficiency and the antioxidant stoichiometry *n*, defined as the number of hypervalent iron species reduced per antioxidant molecule. For all four antioxidants (the two pigments and their chalcones), excellent curve-fittings (*r* > 0.999) were obtained.

From the parameter values (Table 5), the following comments can be made:

(1) the antioxidant efficiency, which lies in the range 10–100, does not allow a clear discrimination between antioxidants. Its drift toward lower values when the antioxidant concentration increases suggests that modelling an antioxidant (stoichiometry *n*) as *n* independent sub-units, each capable of transferring one electron to Fe^IV^ with the same rate constant (*k*_a_), may be too crude and/or that antioxidant–metmyoglobin binding can take place (resulting in two populations of free and bound antioxidant molecules with distinct reactivities).
(2) the antioxidant stoichiometry suggests that a catechol B-ring favors repeated electron transfer to Fe^IV^ (probably through *o*-quinone intermediates) and thus prolonged inhibition. By contrast, the P2 quinonoid base displays a B-ring that is deactivated by the keto group at C4'.
(3) at high antioxidant concentration, the lipid oxidizability tends to decrease. This drift is ascribed to partial heme degradation and to the accumulation of phenolic oxidation products retaining a weak antioxidant character. The latter point is consistent with the structure of P1 oxidation products already determined by us [47].

**Table 5:** Kinetic analysis of the metmyoglobin-induced peroxidation of linoleic acid. Curve-fitting of the *A*(234 nm) *vs.* time curves (CD accumulation). Rate constant of lipid hydroperoxide cleavage by metmyoglobin: *k*_i1_ = 3 × 10^3^ M^−1^·s^−1^ (see Figure 10 & Appendix).

Pigment/μM	*r*_2_/M^−1/2^ s^−1/2^	*AE*	*n*
**P1**, 0.5	2.8 (±0.1)	137 (±16)	3.0 (±0.1)
1	2.6 (±0.1)	40 (±2)	2.5 (±0.1)
1.5	2.3 (±0.1)	38 (±5)	2.5 (±0.1)
2	2.2 (±0.1)	29 (±3)	3.2 (±0.2)
2.5	2.1 (±0.1)	11 (±1)	4.0 (±0.3)
**P1-C_E_**, 0.5	2.7 (±0.1)	108 (±6)	4.4 (±0.1)
1	2.4 (±0.1)	59 (±3)	5.6 (±0.1)
1.5	2.3 (±0.1)	40 (±2)	4.3 (±0.1)
2	2.1 (±0.1)	29 (±1)	5.2 (±0.1)
2.5	1.9 (±0.1)	28 (±1)	3.9 (±0.1)
**P2**, 1.5	2.4 (±0.1)	95 (±6)	0.9 (±0.1)
2.5	2.3 (±0.1)	76 (±14)	0.5 (±0.1)
5	1.9 (±0.1)	15 (±2)	1.4 (±0.1)
6.25	1.6 (±0.1)	17 (±2)	1.2 (±0.1)
7.5	1.0 (±0.1)	24 (±1)	0.9 (±0.1)
**P2-C_E_**, 1.25	2.5 (±0.1)	31 (±3)	3.1 (±0.1)
2.5	2.1 (±0.1)	19 (±1)	3.5 (±0.2)
3.75	1.6 (±0.1)	21 (±2)	1.9 (±0.1)
5	1.2 (±0.1)	29 (±2)	1.3 (±0.1)

## 3. Experimental Section

### 3.1. Chemicals

FeSO_4_, 7H_2_O (98%) and CH_3_CO_2_Na, 3H_2_O (99%) were purchased from Alfa-Aesar. Fe (NO_3_)_3_ (99%) was from Acros. HSA (fraction V, 96%–99%, MW = 66,500 g·mol^−1^), Na_2_HPO_4_, 7H_2_O, NaH_2_PO_4_, 2H_2_O, polyoxyethyleneglycol 23 lauryl ether (Brij^®^35), (9Z, 12Z)-octadecadienoic acid (linoleic acid >99%), myoglobin from equine heart (type ІІ, MW *ca.* 17,600 g·mol^−1^) were from Sigma-Aldrich. Phosphate and acetate buffers were prepared with non-mineralized water C-23597 405 purchased from VWR to limit metal contamination. 3',4',7-Trihydroxyflavylium (P1) and its 7-*O*-β-d-glucoside (P2) were chemically synthesized as described in our previous work [31].

### 3.2. UV-Spectroscopy

An Agilent 8453 UV-visible spectrometer equipped with a 1024-element diode-array detector was used to record the absorption spectra over the wavelength range 190–1100 nm. A water thermostated bath was used to control the cell temperature with an accuracy of ±0.1 °C. The spectroscopic measurements were carried out with a quartz cell of 1 cm optical path length.

### 3.3. Fluorescence Spectroscopy

Steady-state fluorescence spectra were recorded on a thermostated *Safas Xenius* fluorimeter. The excitation and emission slit widths were set at 10 nm. All studies were performed at 37 (±1) °C, excitation at 295 nm (HSA Trp residue), emission light collected between 270 and 410 nm.

### 3.4. Iron-Pigment Binding

To 2 mL of 0.1 M acetate buffer at pH 4.0 placed into the spectrometer cell at 25 °C were successively added 50 µL of a freshly prepared 2 mM pigment solution in acidified MeOH (0.1 M HCl) and 50 µL of freshly prepared iron solution in 0.05 M HCl (concentration range: 1–10 mM). The final iron/pigment molar ratios were in the range 0.5–5. Spectra were typically recorded every 0.5 s over 2 min (binding kinetics) or every 15 s over 15 min (complex stability).

### 3.5. Inhibition of the Heme-Induced Peroxidation of Linoleic Acid

The experimental conditions used were adapted from an already published procedure [21]. Metmyoglobin (17.6 mg) was dissolved in 20 mL of phosphate buffer (20 mM, pH 6.8). After filtration through 0.45 µm filter, its concentration was standardized at 50 µM using ε = 7700 M^−1^·cm^−1^ at 525 nm. Given volumes (20 µL) of daily prepared solutions of linoleic acid (70 mM) in MeOH and pigment (0.05–0.25 mM) were added to 2 mL of Brij^®^35 (4 mM) solution in phosphate buffer (20 mM, pH 5.8). The concentrated solutions of pigments were (a) prepared in 0.1 M HCl in MeOH for investigating inhibition by the colored forms or (b) incubated in the buffer for 24 h at 37 °C to ensure maximal conversion into the corresponding chalcones. The non-ionic surfactant Brij^®^35 was chosen for its good stability and very low content of hydroperoxides, which could react with iron. The final concentrations in the cell were 0.7 mM linoleic acid and 0.5–2.5 μM pigment. Oxidation was initiated by adding 20 μL of the 50 μM metmyoglobin solution (final concentration in the cell: 0.5 µM) to the sample under constant magnetic stirring in open air at 37 °C. Each experiment was run in duplicate. Lipid peroxidation was followed by monitoring the concentration of conjugated dienes (CDs) at 234 nm using ε = 24 × 10^3^ M^−1^·cm^−1^.

### 3.6. Influence of HSA on the Structural Transformations of Pigments

Aliquots (50 µL) of 2 mM solution of pigments prepared in acidified MeOH (0.1 M HCl) were added to 2 mL of pH 7.4 phosphate buffer (50 mM Na_2_HPO_4_ + 100 mM NaCl) in the presence or absence of HSA (0–2 equiv.) at 37 °C. Spectra were recorded every 30 s over 7000 s. All experiments were carried out twice.

Similar experiments were also carried out after varying the pH of the phosphate buffer in the range 6–8. The spectra were recorded immediately after pigment addition and after equilibration over *ca*. 24 h.

### 3.7. Pigment-HSA Binding

Solutions were prepared daily by dissolving HSA in a pH 7.4 buffer (50 mM phosphate + 100 mM NaCl). Aliquots of a 0.5 mM (P1) or 2 mM (P2) solutions were added via syringe to 2 mL of a 2 µM HSA solution placed in a quartz cell (path length: 1 cm) at 37 °C. The concentrated solutions of pigments were (a) prepared in 0.1 M HCl in MeOH for investigating flavylium–HSA binding (MeOH concentration ≤2.5%) or (b) incubated in the buffer for 24 h at 37 °C to ensure maximal conversion into the corresponding chalcones.

For investigating flavylium-HSA binding, a single addition was carried out with subsequent recording of the fluorescence spectrum and renewal of the sample for the next pigment concentration. In such conditions, the flavylium-to-chalcone conversion is negligible.

### 3.8. Data Analysis

All curve-fittings were carried out with the Scientist software (MicroMath, Salt Lake City, UT, USA) through least square regression. They yielded optimized values for the parameters implemented in the models (see Text & Appendix). Standard deviations are reported.

## 4. Conclusions

In this work, 3',4',7-trihydroxyflavylium chloride (P1) and its more water-soluble 7-*O*-β-d-glucopyranoside (P2), come up as suitable models for investigating important properties of anthocyanins: binding of iron ions and serum albumin, inhibition of lipid peroxidation induced by dietary iron in model gastric conditions.

Binding of Fe^III^ is typically fast, especially with the glucoside, and promotes both color variation (due to B-ring deprotonation and additional ligand-to-iron charge transfer) and stabilization (due to the quenching of chalcone formation). Binding of Fe^II^ by itself is not detectable at pH 4 but both pigments promote Fe^II^ autoxidation (followed by the binding of Fe^III^ thus formed), a phenomenon that can be considered protective as Fe^II^ is a potential pro-oxidant through the Fenton reaction. Here again, the glucoside appears superior in accelerating Fe^II^ autoxidation, so that the competing chalcone formation is barely detectable. Binding of serum albumin is weaker with the glucoside, probably because of steric repulsion. It is noteworthy that the chalcone forms a good HSA ligand. In particular, the chalcone glucoside binds HSA three times more tightly than the corresponding colored forms. Consequently, our study suggests that 3-deoxyanthocyanins could partly circulate under their chalcone form in the blood.

Finally, the chalcone forms appear as better inhibitors of heme-induced lipid peroxidation, especially in the case of the glucoside (poorly reactive in its colored form). This prevailing role of the colorless forms in the antioxidant protection afforded by anthocyanins is original and probably important as the physical conditions occurring in the GI tract (temperature, pH, interactions with dietary proteins) could well favor the conversion of the colored forms into the colorless forms.

Overall, 3-deoxyanthocyanins and their chalcones are potentially attractive colorants and antioxidants. Their stability and accessibility by chemical synthesis could foster industrial developments. For instance, iron–3-deoxyanthocyanin chelates could be used in the preparation of colored gels for applications in the food and cosmetic industries [48]. 3-Deoxyanthocyanins could also be developed as natural pH indicators, e.g., for food packaging [49]. They deserve additional investigation of their health-related properties (e.g., their bioavailability).

## Supplementary Materials

Supplementary materials can be accessed at: http://www.mdpi.com/1420-3049/19/12/20709/s1.

## Appendix

### Mathematical Treatment for the Inhibition of Heme-Induced Lipid Peroxidation

The reactions and the corresponding rate constants are displayed in Figure 10.

The peroxidation rate can be written as:

*R_p_ = d(LOOH)/dt = k_p_(LOO^•^)(LH) − k_i1_(LOOH)(Fe^III^) − k_i2_(LOOH)(Fe^IV^) = R_p_ − R_i1_ − R_i2_* 

The rate of lipid consumption is: *−d(LH)/dt = R_p_*

The rate of antioxidant consumption is: *R_a_ =* −*d(AH)/dt*

Assuming a steady-state for the lipid peroxyl radicals, we may write: *R_i2_ = 2k_t_(LOO^•^)^2^*

We thus deduce: $R_{p}=r_{2}\text{(}LH\text{)}R_{\text{i2}}^{\text{1/2}}-R_{i1}-R_{i2}$ with *r_2_ = k_p_/(2k_t_)^1/2^*

Finally, one has: −*d(Fe^III^)/dt = d(Fe^IV^)/dt = R_i1_* − *R_i2_* − *R_a_*

In the absence of antioxidant, the short lag phase is better reproduced without assuming a steady-state for Fe^IV^. On the other hand, the two initiation rate constants can be taken equal (*k_i1_* = *k_i2_*) so as to restrict the total number of adjustable parameters. We thus estimate *k*_i1_ (rate constant of LOOH cleavage by low-valence heme): *k*_i1_ = 3 × 10^3^ M^−1^·s^−1^.

In the presence of an antioxidant, a steady-state for Fe^IV^ can be assumed: *R_i1_ = R_i2_ + R_a_*

This relationship can be written as: *k_i1_(LOOH)(Fe^III^) = [k_i2_(LOOH) + k_a_(AH)](Fe^IV^)*

We thus deduce: $R_{i2}=\frac{R_{i1}}{1+\frac{AE(AH)}{\left(LOOH\right)}}$ with *AE = k_a_/k_i2_* (antioxidant efficiency at inhibiting initiation).

Using the *k*_i1_ value previously determined, the curves of inhibited lipid peroxidation are analyzed to estimate the oxidizability *r*_2_, antioxidant efficiency *AE* and stoichiometry *n*. Parameter *n* is defined as the number of hypervalent iron species reduced per antioxidant molecule. It is implemented in the program by the following initial condition: AH concentration = *n* × total antioxidant concentration.
