# Supplementary material for: Chemically Synthesized Glycosides of Hydroxylated Flavylium Ions as Suitable Models of Anthocyanins: Binding to Iron Ions and Human Serum Albumin, Antioxidant Activity in Model Gastric Conditions
Source: Molecules. 2014 Dec 11;19(12):20709–30. doi: 10.3390/molecules191220709 (PMC6271493; doi:10.3390/molecules191220709)

Supplementary Materials

Figure S1. Time dependence of the A(470 nm) (■) and A(650 nm) (●) after addition of Fe^{II} (5 equiv.) to a P2 solution (pH 4 acetate buffer, 25 °C, pigment concentration = 50 μM).

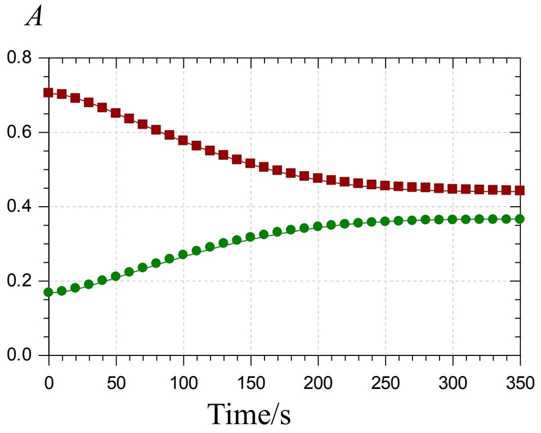

Figure S2. Spectroscopic titration of P1 in the absence (■, 550 nm) or presence (●, 580 nm, 5 equiv.) of HSA. (**A**) spectra recorded immediately after P1 addition, (**B**) spectra recorded after equilibration over *ca*. 24 h.

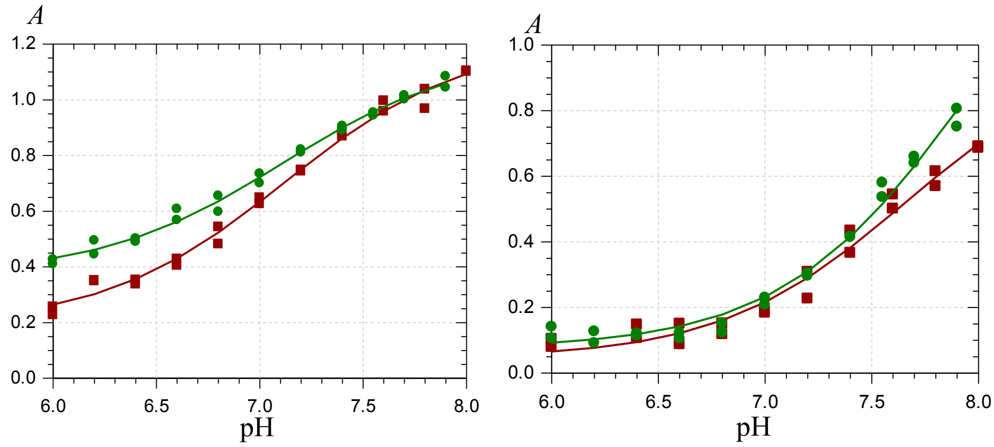

(**A**) (**B**)

Figure S3. Distribution diagram of P1 species at equilibrium around neutrality (in the presence or absence of HSA): neutral quinonoid bases (▬), anionic quinonoid base (▬), chalcone (▬).

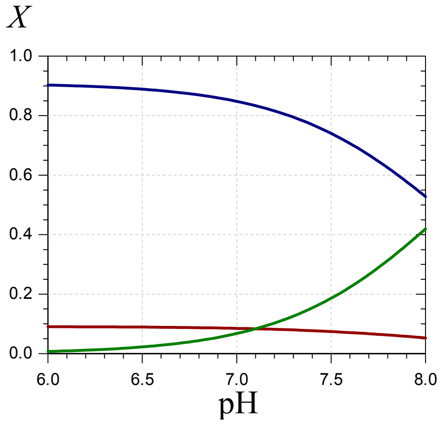

Supplement: Supplementary file 1 [file molecules-19-20709-s001.pdf]
